# Neutrophil Percentage to Albumin Ratio was Associated with Clinical Outcomes in Coronary Care Unit Patients

**DOI:** 10.31083/j.rcm2310333

**Published:** 2022-09-28

**Authors:** Chenghui Cai, Biyang Zhang, Tienan Sun, Fang Zhao, Jun Ma, Xin Pei, Chen He, Hao Che, Liyun Zhao, Yun Wang

**Affiliations:** ^1^Anesthesiology Department, Beijing Anzhen Hospital Affiliated to Capital Medical University, 100029 Beijing, China; ^2^Anesthesiology Department, Beijing Chaoyang Hospital Affiliated to Capital Medical University, 100020 Beijing, China; ^3^Cardiology Department, Beijing Anzhen Hospital Affiliated to Capital Medical University, 100029 Beijing, China

**Keywords:** coronary care unit, neutrophil percentage to albumin ratio, in-hospital mortality, acute kidney injury, predictive ability

## Abstract

**Background:**

Neutrophil percentage to albumin ratio (NPAR) has been shown to be correlated with the prognosis of various diseases. This study aimed to explore the effect of NPAR on the prognosis of patients in coronary care units (CCU).

**Method:**

All data in this study were extracted from the Medical Information Mart for Intensive Care III (MIMIC-III, version1.4) database. All patients were divided into four groups according to their NPAR quartiles. The primary outcome was in-hospital mortality. Secondary outcomes were 30-day mortality, 365-day mortality, length of CCU stay, length of hospital stay, acute kidney injury (AKI), and continuous renal replacement therapy (CRRT). A multivariate binary logistic regression analysis was performed to confirm the independent effects of NPAR. Cox regression analysis was performed to analyze the association between NPAR and 365-day mortality. The curve in line with overall trend was drawn by local weighted regression (Lowess). Subgroup analysis was used to determine the effect of NPAR on in-hospital mortality in different subgroups. Receiver operating characteristic (ROC) curves were used to evaluate the ability of NPAR to predict in-hospital mortality. Kaplan–Meier curves were constructed to compare the cumulative survival rates among different groups.

**Result:**

A total of 2364 patients in CCU were enrolled in this study. The in-hospital mortality rate increased significantly as the NPAR quartiles increased (*p *< 0.001). In multivariate logistic regression analysis, NPAR was independently associated with in-hospital mortality (quartile 4 versus quartile 1: odds ratio [OR], 95% confidence interval [CI]: 1.83, 1.20–2.79, *p* = 0.005, *p* for trend <0.001). In Cox regression analysis, NPAR was independently associated with 365-day mortality (quartile 4 versus quartile 1: OR, 95% CI: 1.62, 1.16–2.28, *p* = 0.005, *p* for trend <0.001). The Lowess curves showed a positive relationship between NPAR and in-hospital mortality. The moderate ability of NPAR to predict in-hospital mortality was demonstrated through ROC curves. The area under the curves (AUC) of NPAR was 0.653 (*p *< 0.001), which is better than that of the platelet to lymphocyte ratio (PLR) (*p *< 0.001) and neutrophil count (*p *< 0.001) but lower than the Sequential Organ Failure Assessment (*p* = 0.046) and Simplified Acute Physiology Score II (*p *< 0.001). Subgroup analysis did not reveal any obvious interactions in most subgroups. However, Kaplan–Meier curves showed that as NPAR quartiles increased, the 30-day (log-rank, *p *< 0.001) and 365-day (log-rank, *p *< 0.001) cumulative survival rates decreased significantly. NPAR was also independently associated with AKI (quartile 4 versus quartile 1: OR, 95% CI: 1.57, 1.19–2.07, *p* = 0.002, *p* for trend = 0.001). The CCU and hospital stay length was significantly prolonged in the higher NPAR quartiles.

**Conclusions:**

NPAR is an independent risk factor for in-hospital mortality in patients in CCU and has a moderate ability to predict in-hospital mortality.

## 1. Introduction

In the past few decades, cardiovascular disease has remained a leading cause of death worldwide, despite a great improvement in prognosis [1, 2]. In this case, a coronary care unit (CCU) was established to focus on managing patients with cardiovascular diseases who may require meticulous care and targeted treatment to reduce adverse outcomes [3, 4, 5]. Clinicians never ceased to explore prognostic indicators that are cheap and available for patients in CCU.

Inflammatory factors are closely associated with the occurrence and development of many cardiovascular diseases [6]. For example, as a major player in acute inflammatory responses, a higher neutrophil percentage was associated with increased mortality risk among patients with acute coronary syndrome [7]. Similarly, Gupta *et al*. [8] confirmed that serum albumin levels play an independent prognostic role in patients with acute and chronic diseases. The neutrophil percentage to albumin ratio (NPAR), a combination of two classical clinical evaluation parameters, was calculated by dividing the neutrophil percentage by the serum albumin concentration and has now become a novel prognostic marker. Previous studies have shown that NPAR is closely associated with the prognosis of severe sepsis and acute kidney injury (AKI) [9, 10]. Moreover, increased NPAR is associated with higher in-hospital mortality and reinfarction rates in patients with ST-elevation myocardial infarction (STEMI) [11]. However, no study has shown a correlation between NPAR and worse outcomes in patients in CCU. From this perspective, this study was based on the hypothesis that NPAR can be considered an independent predictor of adverse events in patients admitted to the CCU.

## 2. Method

### 2.1 Data Source

We extracted all data from an openly available critical care database named Medical Information Mart for Intensive Care III (MIMIC-III, version 1.4) [12], which included data of over 60000 intensive care unit (ICU) stays and over 50000 stays for adult patients. The data in MIMIC-III were collected from June 2001 to October 2012 at the Beth Israel Deaconess Medical Center, including general information (patient demographics, birth and death, and ICU admission and discharge information), vital signs, laboratory data, balance of body fluids, reports, medication, and nursing records. The Protecting Human Research Participants examination was passed to gain access to the MIMIC-III database, and our certificate number is 36571208.

### 2.2 Study Population

**Fig. 1.**
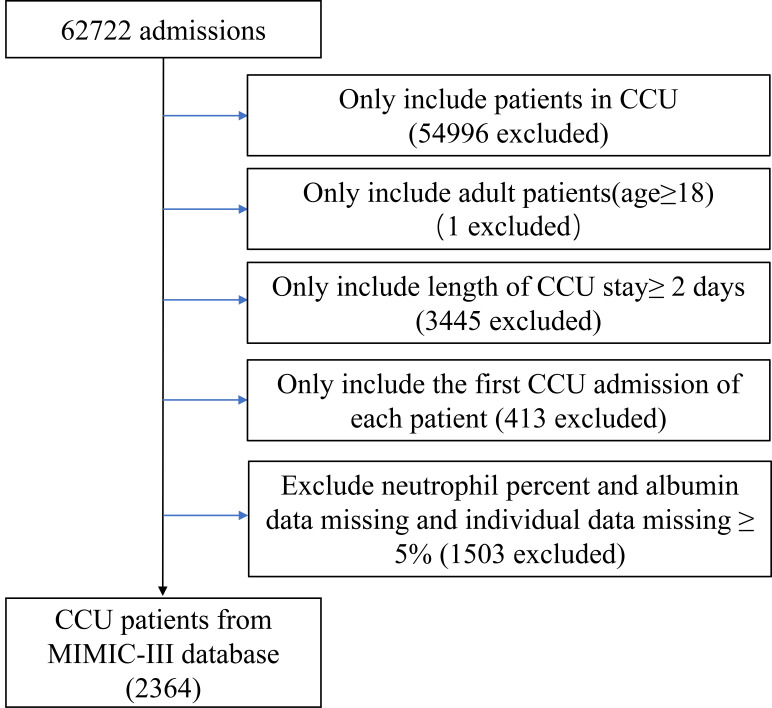
**Flow chart of study population**. CCU, coronary care unit.

All adult patients (≥18 years old) admitted to the CCU were included, and only the first admission of each patient was included. Patients who met the following criteria were excluded: (1) age <18 years; (2) length of CCU stay <2 days; (3) missing neutrophil percentage and albumin data; and (4) missing individual data >5%. Finally, 2364 patients were included in this study (Fig. 1).

### 2.3 Definition of NPAR and Outcomes

NPAR was calculated as the neutrophil percentage divided by the serum albumin concentration. Neutrophil percentage and serum albumin concentration were obtained from the first blood test report after admission to the CCU and measured simultaneously within 24 h. The primary outcome was in-hospital mortality, and the secondary outcomes were 30-day mortality, 365-day mortality, length of CCU stay, length of hospital stay, AKI, and continuous renal replacement therapy (CRRT).

### 2.4 Data Extraction

All data used in this study was extracted using Structured Query Language (SQL) from MIMIC-III database. Demographics, vital signs, diagnoses of heart diseases, comorbidities and medical history, laboratory parameters, medication use, scoring systems (SOFA (sequential organ failure assessment score) [13] and SAPS II (simplified acute physiology score) [14]) and survival data were collected. All laboratory parameters were extracted within 24 hours after admission to CCU.

Demographics were extracted from tables named “admissions” and “patients” of MIMIC-III database. Vital signs were extracted from table named “vitalsfirstday” of MIMIC-III database. Diagnoses of heart diseases, comorbidities and medical history were extracted from table named “diagnoses_icd” of MIMIC-III database. Laboratory parameters were extracted from table named “labevents” of MIMIC-III database. Medication use was extracted from table named “prescriptions” of MIMIC-III database. SOFA and SAPS II were extracted from table named “sofa” and “sapsii” of MIMIC-III database.

### 2.5 Statistical Analysis

All patients in CCU were divided into four groups based on NPAR quartiles. Normally distributed variables are described as mean ± standard deviation (SD), and non-normally distributed variables are described as median interquartile range [IQR]. The differences between the groups were tested using the Kruskal–Wallis test or one-way analysis of variance. Categorical variables are described as numbers (%), and the differences between groups were tested using the chi-square test.

Binary logistic regression analysis was used to analyze the relationship between the NPAR levels and clinical outcomes. Cox regression analysis was performed to analyze the association between NPAR and 365-day mortality. Covariates were included in the regression model based on statistical evidence and clinical judgment. The curves that conformed to the general trend were plotted through local weighted regression (Lowess). Subgroup analysis was used to assess the impact of NPAR on in-hospital mortality in different subgroups. Receiver operating characteristic (ROC) curves were drawn, and areas under the curves (AUC) of different parameters were compared using the DeLong test. The log-rank test was used to compare the 30-day and 365-day survival rates of the different groups, and Kaplan–Meier curves were plotted.

Statistical significance was set at *p *< 0.05, and all tests were two-sided. We used MedCalc (version 15.2.2, Ostend, Belgium) and Stata (v.11.2, 4905 Lakeway Drive, College Station, Texas 77845 USA) for statistical analysis. GraphPad Prism 8 (GraphPad Prism Software Inc., San Diego, CA, USA) was used to draw Kaplan–Meier curves and ROC curves.

## 3. Result

### 3.1 Patient Characteristics

A total of 2364 patients in CCU were enrolled in this study (Fig. 1), and their characteristics stratified using NPAR quartiles were recorded. Of these patients, 576 were included in the first quartile group (NPAR <2.1), 502 were included in the second quartile group (2.1 ≤ NPAR < 2.4), 662 were included in the third quartile group (2.4 ≤ NPAR < 2.9), and 624 patients were included in the fourth quartile group (NPAR ≥2.9). A total of 1357 men and 1007 women were included, most of whom were white. Patients in the highest quartile of NPAR levels had more comorbidities or a history of atrial fibrillation, endocarditis, cardiogenic shock, respiratory failure, sepsis, coronary artery disease, congestive heart failure, primary cardiomyopathy, heart valve disease, hypertension, hypercholesterolemia, and prior myocardial infarction. Moreover, patients in the highest quartile of NPAR levels received less antiplatelet, oral anticoagulant, beta-blocker, angiotensin-converting-enzyme inhibitor/angiotensin II receptor blocker, statin, and diuretics and received more vasopressin treatment. They also had a higher heart rate, respiratory rate, white blood cell, platelet count, blood nitrogen urea, and Sequential Organ Failure Assessment (SOFA) and Simplified Acute Physiology Score (SAPS) II scores but lower blood pressure, lymphocyte, hemoglobin, hematocrit, glucose, and sodium levels (Table 1).

**Table 1.** **Characteristics of patients stratified by NPAR quartiles**.

Characteristics		Total	Quartiles of NPAR				*p* Value
		(n = 2364)	Quartile 1 (n = 576)	Quartile 2 (n = 502)	Quartile 3 (n = 662)	Quartile 4 (n = 624)	
			NPAR <2.1	2.1 ≤ NPAR < 2.4	2.4 ≤ NPAR < 2.9	NPAR ≥2.9	
Age (years)		68.6 ± 14.9	66.1 ± 15.6	69.3 ± 14.5	69.6 ± 14.6	69.1 ± 14.5	<0.001
Gender, n (%)							0.188
	Male	1357 (57.4)	336 (58.3)	305 (60.8)	376 (56.8)	340 (54.5)	
	Female	1007 (42.6)	240 (41.7)	197 (39.2)	286 (43.2)	284 (45.5)	
Race, n (%)							<0.001
	White	1696 (71.7)	417 (72.4)	367 (73.1)	489 (73.9)	423 (67.8)	
	Black	205 (8.7)	68 (11.8)	37 (7.4)	54 (8.2)	46 (7.4)	
	Other	463 (19.6)	91 (15.8)	98 (19.5)	119 (18.0)	155 (24.8)	
Body mass index (kg/$m^{2}$)		28.0 ± 6.5	28.6 ± 6.9	28.3 ± 6.3	27.9 ± 6.3	27.4 ± 6.6	0.009
Vital signs							
	Systolic blood pressure (mmHg)	114.1 ± 16.7	116.2 ± 17.0	114.1 ± 16.6	114.6 ± 16.9	111.7 ± 16.1	<0.001
	Diastolic blood pressure (mmHg)	58.6 ± 10.8	60.6 ± 11.3	58.9 ± 10.7	58.6 ± 10.7	56.7 ± 10.1	<0.001
	Mean blood pressure (mmHg)	76.0 ± 11.0	77.9 ± 11.4	75.9 ± 10.9	76.0 ± 11.1	74.3 ± 10.5	<0.001
	Heart rate (beats/min)	84.6 ± 16.7	82.5 ± 16.6	82.0 ± 15.6	84.2 ± 16.0	89.2 ± 17.5	<0.001
	Respiratory rate (beats/min)	19.5 ± 4.1	19.1 ± 3.9	19.3 ± 3.9	19.6 ± 4.2	19.9 ± 4.4	0.004
	Temperature (°C)	36.8 ± 0.7	36.8 ± 0.6	36.7 ± 0.7	36.7 ± 0.8	36.8 ± 0.8	0.017
	Oxygen saturation (%)	97.1 ± 2.2	97.1 ± 1.8	97.1 ± 2.0	97.1 ± 2.1	97.1 ± 2.9	0.887
Diagnoses of heart diseases, n (%)							
	Coronary artery disease	1058 (44.8)	257 (44.6)	261 (52.0)	319 (48.2)	221 (35.4)	<0.001
	Acute myocardial infarction	356 (15.1)	75 (13.0)	86 (17.1)	105 (18.9)	90 (14.4)	0.252
	Atrial fibrillation	926 (39.2)	197 (34.2)	202 (40.2)	269 (40.6)	258 (41.4)	0.045
	Ventricular arrhythmias	129 (5.5)	22 (3.8)	30 (6.0)	46 (7.0)	31 (5.0)	0.094
	Third-degree atrioventricular block	87 (3.7)	30 (5.2)	17 (3.4)	24 (3.6)	16 (2.6)	0.106
	Congestive heart failure	1347 (57.0)	319 (55.4)	313 (62.4)	387 (58.5)	328 (52.6)	0.007
	Primary cardiomyopathy	210 (8.9)	77 (13.4)	46 (9.2)	52 (7.9)	35 (5.6)	<0.001
	Valve disease	534 (22.6)	136 (23.6)	137 (27.3)	149 (22.5)	112 (18.0)	0.002
	Endocarditis	60 (2.5)	9 (1.6)	4 (0.8)	14 (2.1)	33 (5.3)	<0.001
	Cardiogenic shock	337 (14.3)	53 (9.2)	73 (14.5)	106 (16.0)	105 (16.8)	0.001
Comorbidities and medical history, n (%)							
	Hypertension	866 (36.6)	249 (43.2)	185 (36.9)	252 (38.1)	180 (28.9)	<0.001
	Diabetes	844 (35.7)	204 (35.4)	180 (35.9)	260 (39.3)	200 (32.1)	0.062
	Hypercholesterolemia	695 (29.4)	212 (36.8)	153 (30.5)	199 (30.1)	131 (21.0)	<0.001
	Chronic lung disease	582 (24.6)	118 (20.5)	136 (27.1)	282 (27.5)	146 (23.4)	0.015
	Respiratory failure	603 (25.5)	85 (14.8)	112 (22.3)	195 (29.5)	211 (33.8)	<0.001
	Chronic kidney disease	552 (23.4)	118 (20.5)	127 (25.3)	171 (25.8)	136 (21.8)	0.078
	Chronic liver disease	106 (4.5)	27 (4.7)	20 (4.0)	28 (4.2)	31 (5.0)	0.852
	Malignancy	343 (14.5)	71 (12.3)	65 (13.0)	107 (16.2)	100 (16.0)	0.121
	Autoimmune disease	122 (5.2)	19 (3.3)	24 (4.8)	40 (6.0)	39 (6.3)	0.079
	Sepsis	318 (14.5)	43 (7.5)	45 (9.0)	92 (13.9)	138 (22.1)	<0.001
	Prior myocardial infarction	199 (8.4)	44 (7.6)	50 (10.0)	69 (10.4)	36 (5.8)	0.011
	Prior stroke	54 (2.3)	10 (1.7)	15 (3.0)	19 (2.9)	10 (1.6)	0.240
Laboratory parameters							
	Neutrophil (%)	78.6 ± 12.2	66.2 ± 14.3	78.5 ± 8.5	82.8 ± 7.6	85.8 ± 6.7	<0.001
	Albumin (g/L)	32.1 ± 6.1	37.7 ± 4.9	34.8 ± 3.8	31.6 ± 3.2	25.2 ± 3.8	<0.001
	White blood cell (${\mathrm{10}}^{9}$/L)	11.9 ± 6.1	9.9 ± 5.3	11.1 ± 4.9	12.2 ± 5.8	14.2 ± 7.0	<0.001
	Lymphocyte (%)	12.8 ± 8.6	21.4 ± 10.0	12.9 ± 6.4	10.0 ± 5.6	7.8 ± 4.9	<0.001
	Platelet (${\mathrm{10}}^{9}$/L)	238.7 ± 103.6	227.8 ± 94.8	231.4 ± 93.0	236.8 ± 98.4	256.5 ± 121.4	<0.001
	Hemoglobin (g/dL)	11.2 ± 2.0	11.6 ± 2.1	11.7 ± 2.0	11.2 ± 1.9	10.5 ± 1.9	<0.001
	Hematocrit (%)	33.8 ± 5.9	34.7 ± 6.1	35.0 ± 6.0	33.8 ± 5.6	32.0 ± 5.6	<0.001
	Glucose (mg/dL)	156.0 ± 78.9	145.7 ± 71.1	156.3 ± 76.5	164.3 ± 83.2	156.3 ± 81.9	<0.001
	Creatinine (mg/dL)	1.8 ± 1.6	1.7 ± 1.6	1.8 ± 1.7	1.9 ± 1.7	1.8 ± 1.5	0.231
	Blood nitrogen urea (mg/dL)	34.2 ± 23.4	30.1 ± 21.1	34.4 ± 23.8	35.3 ± 23.2	36.9 ± 24.7	<0.001
	Sodium (mmol/L)	137.9 ± 5.0	138.5 ± 4.1	137.8 ± 4.8	137.8 ± 4.6	137.4 ± 6.0	<0.001
	Potassium (mmol/L)	4.3 ± 0.8	4.2 ± 0.8	4.3 ± 0.8	4.3 ± 0.8	4.2 ± 0.8	0.058
	NPAR	2.46 (2.11, 2.93)	1.84 (1.63, 1.98)	2.25 (2.18, 2.33)	2.62 (2.44, 2.75)	3.32 (3.08, 3.69)	<0.001
	PLR	194 (122, 317)	129 (87, 190)	186 (123, 281)	223 (144, 365)	267 (162, 514)	<0.001
	NLR	7.5 (4.3, 13.1)	3.4 (2.3, 5.1)	6.5 (4.3, 10.1)	9.2 (6.3, 14.4)	12.5 (8.0, 22.5)	<0.001
Medication use, n (%)							
	Antiplatelet	1348 (57.0)	327 (56.8)	315 (62.8)	404 (61.0)	302 (48.4)	<0.001
	Oral anticoagulants	713 (30.2)	171 (29.7)	167 (33.3)	212 (32.0)	163 (26.1)	0.040
	Beta-blockers	1619 (68.5)	392 (68.1)	371 (73.9)	473 (71.5)	383 (60.4)	<0.001
	ACEI/ARB	1162 (49.2)	324 (56.3)	280 (55.8)	338 (51.1)	220 (35.3)	<0.001
	Statin	1300 (55.0)	315 (54.7)	317 (63.2)	382 (57.7)	286 (45.8)	<0.001
	Vasopressin	210 (8.9)	33 (5.7)	31 (6.2)	60 (9.1)	86 (13.8)	<0.001
	CCB	751 (31.8)	170 (29.5)	160 (31.9)	210 (31.7)	211 (33.8)	0.465
	Diuretics	1817 (76.9)	436 (75.7)	411 (81.9)	510 (77.0)	460 (73.7)	0.012
Scoring systems							
	SOFA	4 (2, 7)	3 (2, 5)	4 (2, 6)	4 (2, 7)	5 (3, 7)	<0.001
	SAPS II	38 (30, 48)	34 (26, 43)	37 (30, 45)	38 (30, 48)	43 (34, 52)	<0.001

Continuous variables were presented as mean ± SD or median (IQR). Categorical variables were presented as number (percentage). Abbreviation: NPAR, neutrophil percentage to albumin ratio; PLR, platelet to lymphocyte ratio; NLR, neutrophil to lymphocyte ratio; ACEI, angiotensin-converting enzyme inhibitor; ARB, angiotensin receptor blocker; CCB, Calcium channel blocker; SOFA, sequential organ failure assessment score; SAPS II, simplified acute physiology score.

### 3.2 Outcomes

As shown in Table 2, the in-hospital mortality rate of all patients in this study was 16.5%. As NPAR quartiles increased, in-hospital mortality increased gradually (quartile 1 versus quartile 4: 8.3% versus 26.6%, *p *< 0.001); in univariate logistic regression analysis, the risk of in-hospital mortality increased significantly as NPAR quartiles increased (quartile 4 versus quartile 1: odds ratio [OR], 95% confidence interval [CI]: 3.99, 2.82–5.63, *p *< 0.001, *p* for trend <0.001). When examined as a continuous variable in Model 1, for each unit increase in NPAR, the risk of in-hospital mortality increased by 89%. After adjusting for age, sex, and race in Model 2, we reached a similar conclusion. In the multivariate logistic regression analysis, more confounding variables were included. The association between in-hospital mortality and NPAR was attenuated but remained statistically significant (quartile 4 versus quartile 1: OR, 95% CI: 1.83, 1.20–2.79, *p* = 0.005, *p* for trend <0.001). When examined as a continuous variable in Model 3, NPAR was still independently associated with the risk of in-hospital mortality in patients in CCU (OR, 95% CI: 1.24, 1.09–1.42, *p* = 0.001) (Table 3). The direct effect of NPAR on 365-day mortality was confirmed using the Cox regression analysis. After the data were adjusted for potential confounding variables, a positive correlation was observed between NPAR and in-hospital mortality (quartile 4 versus quartile 1: HR, 95% CI: 1.62, 1.16–2.28, *p* = 0.005, *p* for trend <0.001) (Table 4). All variables were proven to have no collinearity relationship in the collinearity test before they were included in the model. Besides, we found that most of the covariables had a linear relationship with the outcome through the Lowess curve, suggesting that the model might have good accuracy and authenticity in clinical practice.

**Table 2.** **Outcomes of patients stratified by NPAR quartiles**.

Outcomes	Total	Quartiles of NPAR				*p* Value
	(n = 2364)	Quartile 1 (n = 576)	Quartile 2 (n = 502)	Quartile 3 (n = 662)	Quartile 4 (n = 624)	
		NPAR <2.1	2.1 ≤ NPAR < 2.4	2.4 ≤ NPAR < 2.9	NPAR ≥2.9	
In-hospital mortality, n (%)	389 (16.5)	48 (8.3)	54 (10.8)	121 (18.3)	166 (26.6)	<0.001
30-day mortality, n (%)	425 (18.0)	51 (8.9)	63 (12.6)	139 (21.0)	172 (27.6)	<0.001
365-day mortality, n (%)	918 (38.8)	135 (23.4)	173 (34.5)	272 (41.1)	338 (54.2)	<0.001
Length of CCU stay (days)	4.5 (3.0, 8.1)	3.6 (2.7, 5.7)	4.2 (3.0, 7.2)	4.6 (3.0, 7.8)	6.4 (3.3, 11.8)	<0.001
Length of hospital stay (days)	10.6 (6.8, 17.7)	8.2 (5.6, 13.4)	10.0 (6.5, 15.3)	10.6 (6.9, 17.2)	13.9 (8.5, 24)	<0.001
Acute kidney injury, n (%)	1416 (59.9)	299 (51.9)	288 (57.4)	412 (62.2)	417 (66.8)	<0.001
Renal replacement therapy, n (%)	294 (12.4)	58 (10.1)	51 (10.2)	92 (13.9)	93 (14.9)	0.017

Non-normally distributed continuous variables were presented as median (IQR). Categorical variables were presented as number (percentage). Abbreviation: NPAR, neutrophil percentage to albumin ratio; CCU, coronary care unit.

**Table 3.** **The association between NPAR and in-hospital all-cause mortality**.

		NPAR		
		OR (95% CI)	*p* Value	p for trend
Model 1				<0.001
	Quartile 1: NPAR <2.1	Ref		
	Quartile 2: 2.1 ≤ NPAR < 2.4	1.33 (0.88, 2.00)	0.176	
	Quartile 3: 2.4 ≤ NPAR < 2.9	2.46 (1.72, 3.51)	<0.001	
	Quartile 4: NPAR ≥2.9	3.99 (2.82, 5.63)	<0.001	
	Continuous	1.89 (1.64, 2.19)	<0.001	
Model 2				<0.001
	Quartile 1: NPAR <2.1	Ref		
	Quartile 2: 2.1 ≤ NPAR < 2.4	1.24 (0.82, 1.88)	0.298	
	Quartile 3: 2.4 ≤ NPAR < 2.9	2.34 (1.64, 3.35)	<0.001	
	Quartile 4: NPAR ≥2.9	3.77 (2.66, 5.33)	<0.001	
	Continuous	1.88 (1.62, 2.18)	<0.001	
Model 3				<0.001
	Quartile 1: NPAR <2.1	Ref		
	Quartile 2: 2.1 ≤ NPAR < 2.4	1.11 (0.69, 1.80)	0.656	
	Quartile 3: 2.4 ≤ NPAR < 2.9	1.64 (1.08, 2.50)	0.022	
	Quartile 4: NPAR ≥2.9	1.83 (1.20, 2.79)	0.005	
	Continuous	1.24 (1.09, 1.42)	0.001	

Models were derived from binary logistic regression analysis. Model 1: unadjusted. Model 2: adjusted for age, gender, race. Model 3: adjusted for age, gender, race, respiratory rate, temperature, body mass index, coronary heart disease, acute myocardial infarction, atrial fibrillation, ventricular arrhythmias, third-degree atrioventricular block, congestive heart failure, primary cardiomyopathy, valve disease, endocarditis, cardiogenic shock, hypertension, diabetes, respiratory failure, chronic kidney disease, chronic lung disease, malignancy, sepsis, prior myocardial infarction, prior stroke, antiplatelet, oral anticoagulants, CCB, diuretics, statin, AKI, ACEI/ARB, hemoglobin, blood nitrogen urea, hematocrit, sodium, creatinine, SAPS II, SOFA. Abbreviation: NPAR, neutrophil percentage to albumin ratio; AKI, acute kidney injury; CCB, Calcium channel blocker; OR, odds ratio; CI, confidence interval.

**Table 4.** **The association between NPAR and 365-day mortality**.

		NPAR		
		HR (95% CI)	*p* Value	p for trend
Model 1				<0.001
	Quartile 1: NPAR <2.1	Ref		
	Quartile 2: 2.1 ≤ NPAR < 2.4	1.16 (0.79, 1.71)	0.458	
	Quartile 3: 2.4 ≤ NPAR < 2.9	1.76 (1.26, 2.45)	<0.001	
	Quartile 4: NPAR ≥2.9	2.38 (1.72, 3.28)	<0.001	
	Continuous	1.89 (1.64, 2.19)	<0.001	
Model 2				<0.001
	Quartile 1: NPAR <2.1	Ref		
	Quartile 2: 2.1 ≤ NPAR < 2.4	1.17 (0.79, 1.72)	0.440	
	Quartile 3: 2.4 ≤ NPAR < 2.9	1.77 (1.27, 2.48)	<0.001	
	Quartile 4: NPAR ≥2.9	2.36 (1.71, 3.26)	<0.001	
Model 3				<0.001
	Quartile 1: NPAR <2.1	Ref		
	Quartile 2: 2.1 ≤ NPAR < 2.4	1.08 (0.73, 1.61)	0.691	
	Quartile 3: 2.4 ≤ NPAR < 2.9	1.55 (1.10, 2.18)	0.013	
	Quartile 4: NPAR ≥2.9	1.62 (1.16, 2.28)	0.005	

Models were derived from Cox regression analysis. Model 1: unadjusted. Model 2: adjusted for age, gender, race. Model 3: adjusted for age, gender, race, respiratory rate, temperature, body mass index, coronary heart disease, acute myocardial infarction, atrial fibrillation, ventricular arrhythmias, third-degree atrioventricular block, congestive heart failure, primary cardiomyopathy, valve disease, endocarditis, cardiogenic shock, hypertension, diabetes, respiratory failure, chronic kidney disease, chronic lung disease, malignancy, sepsis, prior myocardial infarction, prior stroke, antiplatelet, oral anticoagulants, CCB, diuretics, statin, AKI, ACEI/ARB, hemoglobin, blood nitrogen urea, hematocrit, sodium, creatinine, SAPS II, SOFA. Abbreviation: NPAR, neutrophil percentage to albumin ratio; AKI, acute kidney injury; CCB, Calcium channel blocker; HR, hazard ratio; CI, confidence interval.

**Fig. 2.**
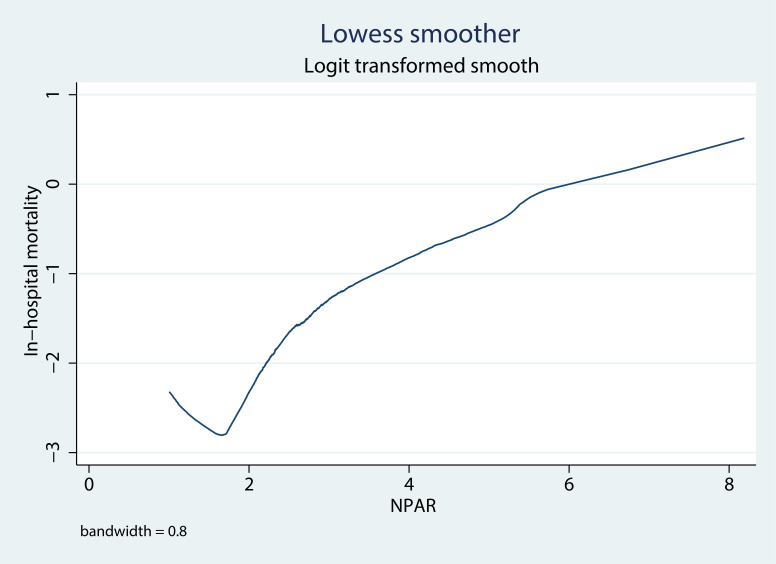
**Association between the NPAR and in-hospital mortality presented through Lowess smoothing**. Abbreviation: NPAR, neutrophil percentage to albumin ratio.

We drew a Lowess curve in our study to explore the association between NPAR and in-hospital mortality (Fig. 2). A non-linear relationship was observed between NPAR and in-hospital mortality. Specifically, when NPAR was less than 1.65, there was a negative correlation between NPAR and mortality. When the NPAR was greater than 1.65, the in-hospital mortality increased as the NPAR increased.

**Fig. 3.**
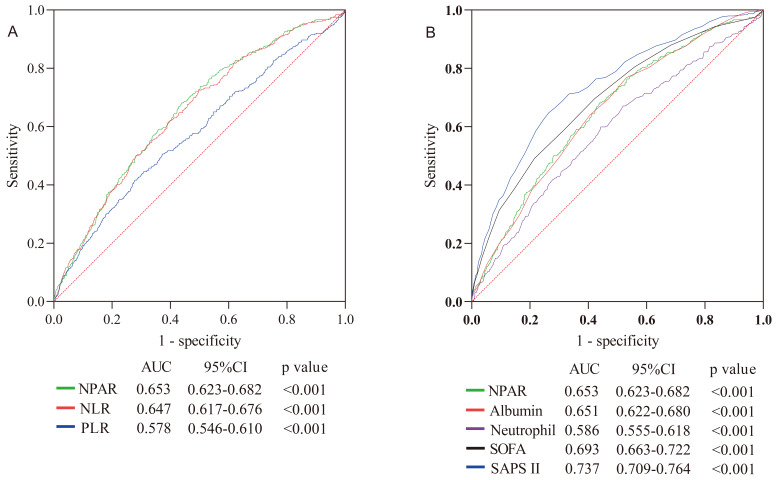
**The ROC curves for the prediction of in-hospital all-cause mortality**. (A) ROC curves for the prediction of in-hospital all-cause mortality of NPAR, NLR, PLR. (B) ROC curves for the prediction of in-hospital all-cause mortality of NPAR, neutrophil, albumin, SOFA, and SAPS II. Abbreviation: NPAR, neutrophil percent to albumin ratio; NLR, neutrophil to lymphocyte ratio; PLR, platelet to lymphocyte ratio; SOFA, sequential organ failure assessment score; SAPS II, simplified acute physiology score.

**Fig. 4.**
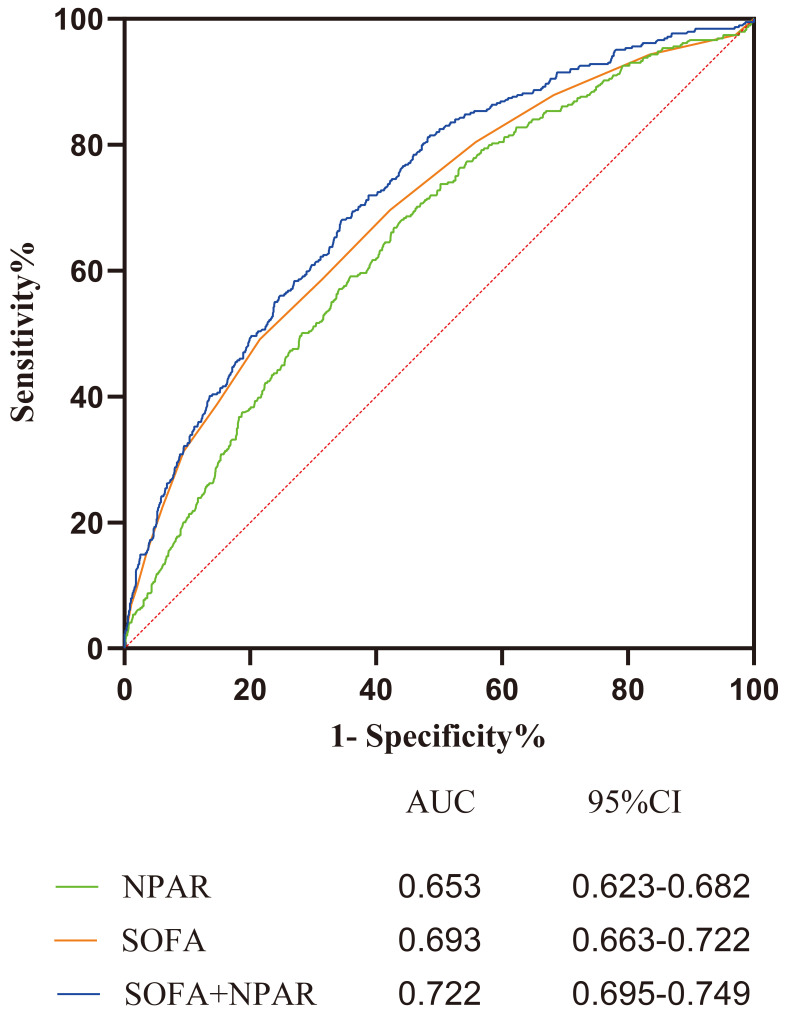
** ROC curves for the prediction of in-hospital all-cause mortality of NPAR, SOFA, NPAR+SOFA**. Abbreviation: NPAR, neutrophil percent to albumin ratio; SOFA, sequential organ failure assessment score.

In the subgroup analysis, no significant interactions were observed in most subgroups. Hypertension, prior myocardial infarction, low glucose, and low blood nitrogen urea enhanced the effect of NPAR on in-hospital mortality. In contrast, cardiogenic shock, respiratory failure, sepsis, vasopressin treatment, and low albumin and sodium levels attenuated the effect of NPAR on in-hospital mortality (Table 5). The ROC curves in Fig. 3 demonstrate that NPAR had a moderate ability to predict in-hospital mortality, with an AUC of 0.653 (*p *< 0.001). Comparing AUCs, the ability of NPAR to predict in-hospital mortality was better than that of platelet to lymphocyte ratio (PLR) (*p *< 0.001) and neutrophil count (*p *< 0.001) but lower than that of SOFA (*p* = 0.046) and SAPS II (*p *< 0.001). No statistical difference was observed between the neutrophil-to-lymphocyte ratio (NLR) (*p* = 0.683) and albumin level (*p* = 0.874). In addition, ROC curves were drawn for NPAR, SOFA, and NPAR+SOFA. We found that when combining NPAR with SOFA, the AUC of 0.722 was obtained, which was larger than the AUC of the two separately, suggesting that the combination of both indices improved the predictive accuracy of adverse outcomes in patients in CCU (Fig. 4).

**Table 5.** **Subgroup analysis of associations between in-hospital all-cause mortality and NPAR**.

Subgroups		N	Quartile 1	Quartile 2	Quartile 3	Quartile 4	*p* for interaction
			NPAR <2.1	2.1≤ NPAR <2.4	2.4≤ NPAR <2.9	NPAR ≥2.9	
Gender							0.074
	Male	1357	Ref	1.57 (0.96, 2.57)	2.25 (1.43, 3.54)	3.49 (2.24, 5.43)	
	Female	1007	Ref	0.85 (0.40, 1.83)	2.85 (1.60, 5.08)	4.88 (2.79, 8.53)	
Age (years)							0.250
	<70	1139	Ref	1.30 (0.69, 2.46)	2.49 (1.44, 4.30)	4.73 (2.82, 7.94)	
	≥70	1225	Ref	1.25 (0.73, 2.15)	2.24 (1.40, 3.58)	3.29 (2.07, 5.23)	
Race							0.367
	White	1696	Ref	1.48 (0.90, 2.43)	2.58 (1.67, 3.99)	4.22 (2.75, 6.46)	
	Black	168	Ref	-	2.38 (0.66, 8.61)	6.30 (1.90, 20.86)	
	Other	463	Ref	1.08 (0.49, 2.42)	2.02 (0.99, 4.15)	2.53 (1.28, 5.00)	
Systolic blood pressure (mmHg)							0.430
	<112	1171	Ref	1.07 (0.64, 1.82)	1.98 (1.25, 3.14)	3.21 (2.07, 4.98)	
	≥112	1193	Ref	1.65 (0.85, 3.21)	3.20 (1.81, 5.67)	4.72 (2.67, 8.34)	
Diastolic blood pressure (mmHg)							0.926
	<57	1109	Ref	1.13 (0.62, 2.06)	1.84 (1.10, 3.10)	3.70 (2.27, 6.05)	
	≥57	1255	Ref	1.49 (0.85, 2.62)	3.10 (1.91, 5.05)	3.96 (2.43, 6.46)	
Mean blood pressure (mmHg)							0.871
	<74	1130	Ref	1.07 (0.59, 1.92)	2.14 (1.29, 3.56)	3.61 (2.23, 5.83)	
	≥74	1234	Ref	1.58 (0.89, 2.79)	2.73 (1.66, 4.49)	3.98 (2.41, 6.58)	
Heart rate (beats/min)							0.835
	<83	1174	Ref	1.46 (0.84, 2.56)	2.90 (1.76, 4.78)	4.17 (2.51, 6.90)	
	≥83	1190	Ref	1.19 (0.65, 2.18)	2.06 (1.25, 3.42)	3.70 (2.29, 5.96)	
Respiratory rate (beats/min)							0.243
	<18	950	Ref	1.15 (0.54, 2.44)	3.15 (1.70, 5.82)	4.74 (2.59, 8.68)	
	≥18	1414	Ref	1.38 (0.85, 2.26)	2.12 (1.37, 3.29)	3.55 (2.33, 5.40)	
Temperature (°C)							0.114
	<36.7	1102	Ref	1.82 (0.99, 3.34)	3.44 (2.00, 5.91)	5.67 (3.34, 9.64)	
	≥36.7	1262	Ref	1.01 (0.57, 1.77)	1.85 (1.15, 2.97)	2.96 (1.87, 4.68)	
Oxygen saturation (%)							0.134
	<97.3	1161	Ref	1.59 (0.88, 2.88)	2.33 (1.36, 3.98)	5.16 (3.08, 8.64)	
	≥97.3	1203	Ref	1.12 (0.63, 1.97)	2.56 (1.59, 4.12)	3.17 (1.99, 5.05)	
Body mass index (kg/$m^{2}$)							0.404
	<27.2	1180	Ref	1.49 (0.85, 2.60)	2.17 (1.32, 3.57)	3.66 (2.28, 5.88)	
	≥27.2	1184	Ref	1.16 (0.64, 2.12)	2.77 (1.67, 4.60)	4.24 (2.56, 7.04)	
Coronary artery disease							0.335
	Yes	1058	Ref	1.51 (0.82, 2.77)	2.90 (1.68, 5.01)	3.29 (1.86, 5.82)	
	No	1306	Ref	1.21 (0.69, 2.11)	2.16 (1.35, 3.47)	4.24 (2.74, 6.57)	
Acute myocardial infarction							0.377
	Yes	356	Ref	0.86 (0.31, 2.41)	2.76 (1.17, 6.49)	2.39 (0.99, 5.80)	
	No	2008	Ref	1.43 (0.91, 2.24)	2.37 (1.60, 3.50)	4.34 (2.98, 6.31)	
Atrial fibrillation							0.379
	Yes	926	Ref	1.19 (0.64, 2.24)	2.76 (1.61, 4.75)	3.23 (1.89, 5.53)	
	No	1438	Ref	1.39 (0.81, 2.39)	2.13 (1.32, 3.42)	4.52 (2.88, 7.09)	
Ventricular arrhythmias							0.348
	Yes	129	Ref	5.00 (0.97, 25.77)	4.38 (0.90, 21.31)	4.76 (0.93, 24.48)	
	No	2235	Ref	1.14 (0.74, 1.75)	2.32 (1.61, 3.35)	3.94 (2.77, 5.61)	
Third-degree atrioventricular block							0.896
	Yes	70	Ref	-	2.31 (0.66, 13.56)	2.52 (0.58, 15.53)	
	No	2277	Ref	1.39 (0.92, 2.11)	2.45 (1.70, 3.53)	4.04 (2.84, 5.76)	
Congestive heart failure							0.789
	Yes	1347	Ref	1.45 (0.86, 2.45)	2.73 (1.71, 4.35)	4.28 (2.70, 6.79)	
	No	1017	Ref	1.11 (0.57, 2.17)	2.08 (1.20, 3.62)	3.68 (2.19, 6.18)	
Primary cardiomyopathy							0.931
	Yes	210	Ref	0.22 (0.03, 1.87)	3.00 (1.09, 8.24)	2.96 (0.98, 8.97)	
	No	2154	Ref	1.47 (0.96, 2.26)	2.43 (1.66, 3.56)	4.10 (2.83, 5.92)	
Valve disease							0.424
	Yes	534	Ref	1.43 (0.61, 3.35)	2.66 (1.23, 5.76)	3.08 (1.39, 6.82)	
	No	1830	Ref	1.30 (0.82, 2.08)	2.40 (1.61, 3.59)	4.14 (2.82, 6.08)	
Endocarditis							0.084
	Yes	60	Ref	2.67 (0.12, 57.62)	4.44 (0.42, 46.54)	1.43 (0.15, 14.05)	
	No	2304	Ref	1.32 (0.87, 1.99)	2.41 (1.68, 3.46)	4.14 (2.92, 5.87)	
Cardiogenic shock							0.021
	Yes	337	Ref	0.50 (0.22, 1.16)	1.35 (0.66,2.73)	1.42 (0.70, 2.88)	
	No	2017	Ref	1.62 (1.00, 2.62)	2.65 (1.73, 4.07)	4.92 (3.26, 7.41)	
Hypertension							0.015
	Yes	866	Ref	1.38 (0.66, 2.89)	3.30 (1.78, 6.10)	6.69 (3.63, 12.33)	
	No	1498	Ref	1.25 (0.76, 2.04)	2.06 (1.33, 3.19)	3.01 (1.98, 4.57)	
Diabetes							0.197
	Yes	844	Ref	0.91 (0.46, 1.78)	1.87 (1.08, 3.26)	2.75 (1.58, 4.80)	
	No	1520	Ref	1.66 (0.99, 2.79)	2.93 (1.84, 4.67)	4.93 (3.16, 7.69)	
Hypercholesterolemia							0.548
	Yes	695	Ref	0.92 (0.40, 2.10)	3.40 (1.82, 6.38)	3.90 (2.01, 7.58)	
	No	1669	Ref	1.45 (0.90, 2.33)	2.10 (1.36, 3.23)	3.82 (2.54, 5.75)	
Chronic lung disease							0.082
	Yes	582	Ref	1.70 (0.70, 4.18)	5.50 (2.50, 12.08)	6.73 (3.04, 14.94)	
	No	1782	Ref	1.25 (0.78, 1.98)	1.75 (1.16, 2.65)	3.43 (2.33, 5.04)	
Respiratory failure							0.005
	Yes	603	Ref	0.66 (0.33, 1.34)	1.38 (0.76, 2.48)	1.55 (0.87, 2.76)	
	No	1761	Ref	1.63 (0.97, 2.73)	2.58 (1.62, 4.10)	5.14 (3.30, 8.02)	
Chronic kidney disease							0.242
	Yes	552	Ref	0.84 (0.37, 1.93)	1.72 (0.85, 3.45)	2.49 (1.23, 5.00)	
	No	1812	Ref	1.52 (0.95, 2.44)	2.75 (1.82, 4.16)	4.57 (3.07, 6.81)	
Malignancy							0.529
	Yes	343	Ref	0.71 (0.19, 2.64)	3.66 (1.42, 9.39)	4.21 (1.64, 10.82)	
	No	2021	Ref	1.42 (0.92, 2.19)	2.25 (1.53, 3.30)	3.94 (2.72, 5.71)	
Autoimmune disease							0.596
	Yes	122	Ref	2.57 (0.25, 26.94)	5.23 (0.61, 44.69)	8.00 (0.96, 67.01)	
	No	2242	Ref	1.30 (0.85, 1.97)	2.38 (1.66, 3.42)	3.88 (2.73, 5.51)	
Sepsis							0.001
	Yes	318	Ref	0.94 (0.38, 2.29)	1.27 (0.59, 2.73)	1.29 (0.63, 2.66)	
	No	2046	Ref	1.41 (0.88, 2.26)	2.61 (1.72, 3.95)	4.45 (2.96, 6.67)	
Prior myocardial infarction							0.034
	Yes	199	Ref	2.74 (0.27, 27.39)	10.95 (1.38, 86.53)	21.50 (2.63, 175.61)	
	No	2165	Ref	1.31 (0.86, 1.99)	2.27 (1.58, 3.27)	3.66 (2.58, 5.20)	
Prior stroke							0.815
	Yes	54	Ref	0.64 (0.04, 11.63)	1.69 (0.15, 18.71)	2.25 (0.17, 29.77)	
	No	2310	Ref	1.35 (0.89, 2.04)	2.48 (1.73, 3.55)	4.02 (2.84, 5.70)	
Antiplatelet							0.053
	Yes	1348	Ref	1.22 (0.75, 2.00)	2.34 (1.52, 3.60)	3.10 (1.99, 4.83)	
	No	1016	Ref	1.46 (0.70, 3.03)	2.61 (1.39, 4.89)	5.87 (3.30, 10.44)	
Oral anticoagulants							0.815
	Yes	713	Ref	1.27 (0.51, 3.15)	2.41 (1.09, 5.30)	3.57 (1.62, 7.86)	
	No	1651	Ref	1.38 (0.87, 2.19)	2.54 (1.71, 3.80)	4.05 (2.76, 5.96)	
Beta-blockers							0.067
	Yes	1619	Ref	1,13 (0.69, 1.85)	2.18 (1.42, 3.33)	2.96 (1.93, 4.53)	
	No	745	Ref	1.93 (0.92, 4.05)	3.26 (1.71, 6.23)	6.26 (3.41, 11.49)	
ACEI/ARB							0.223
	Yes	1162	Ref	1.79 (0.84, 3.78)	3.20 (1.64, 6.25)	4.92 (2.49, 9.71)	
	No	1202	Ref	1.16 (0.70, 1.92)	2.10 (1.36, 3.23)	2.88 (1.91, 4.34)	
Statin							0.665
	Yes	1300	Ref	1.22 (0.69, 2.15)	2.67 (1.64, 4.37)	3.43 (2.07, 5.66)	
	No	1064	Ref	1.54 (0.85, 2.80)	2.25 (1.34, 3.78)	4.33 (2.68, 6.99)	
Vasopressin							0.017
	Yes	210	Ref	0.75 (0.27, 2.05)	1.19 (0.50, 2.80)	1.42 (0.63, 3.19)	
	No	2154	Ref	1.50 (0.94, 2.40)	2.74 (1.81, 4.13)	4.39 (2.94, 6.56)	
White blood cell (${\mathrm{10}}^{9}$/L)							0.442
	<10.6	1166	Ref	1.65 (0.93, 2.91)	2.71 (1.62, 4.51)	4.54 (2.73, 7.56)	
	≥10.6	1198	Ref	0.93 (0.51, 1.68)	1.86 (1.12, 3.08)	2.85 (1.75, 4.63)	
Neutrophil (%)							0.188
	<81	1178	Ref	1.24 (0.77, 2.00)	1.95 (1.22, 3.11)	3.43 (2.10, 5.61)	
	≥81	1186	Ref	3.31 (0.75, 14.50)	6.66 (1.59, 27.85)	10.2 (2.45, 42.39)	
Lymphocyte (%)							0.529
	<11	1178	Ref	0.59 (0.29, 1.19)	1.11 (0.60, 2.05)	1.60 (0.88, 2.91)	
	≥11	1186	Ref	1.46 (0.86, 2.47)	1.99 (1.18, 3.34)	3.51 (2.04, 6.05)	
Platelet ($m^{9}$/L)							0.167
	<221	1175	Ref	1.34 (0.79, 2.30)	2.39 (1.49, 3.84)	3.31 (2.06, 5.32)	
	≥221	1189	Ref	1.30 (0.69, 2.44)	2.59 (1.51, 4.43)	4.86 (2.91, 8.12)	
Hemoglobin (g/dL)							0.925
	<11.1	1174	Ref	1.22 (0.64, 2.34)	2.40 (1.39, 4.15)	3.86 (2.30, 6.48)	
	≥11.1	1190	Ref	1.40 (0.83, 2.37)	2.52 (1.58, 4.03)	4.24 (2.61, 6.89)	
Hematocrit (%)							0.232
	<33.3	1174	Ref	1.07 (0.57, 2.02)	2.28 (1.35, 3.83)	3.22 (1.97, 5.29)	
	≥33.3	1190	Ref	1.54 (0.90, 2.64)	2.62 (1.61, 4.25)	5.25 (3.22, 8.57)	
Glucose (mg/dL)							0.037
	<132	1167	Ref	2.73 (1.50, 4.96)	3.28 (1.86, 5.78)	7.16 (4.19, 12.23)	
	≥132	1197	Ref	0.63 (0.35, 1.14)	1.85 (1.17, 2.93)	2.31 (1.46, 3.65)	
Creatinine (mg/dL)							0.128
	<1.2	1061	Ref	1.12 (0.55, 2.30)	3.04 (1.70, 5.46)	4.95 (2.82, 8.70)	
	≥1.2	1303	Ref	1.38 (0.83, 2.28)	2.07 (1.32, 3.24)	3.37 (2.18, 5.23)	
Blood nitrogen urea (mg/dL)							0.044
	<27	1168	Ref	1.49 (0.74, 2.99)	3.77 (2.10, 6.78)	5.72 (3.22, 10.17)	
	≥27	1196	Ref	1.15 (0.69, 1.91)	1.71 (1.09, 2.69)	2.88 (1.86, 4.47)	
Albumin (g/L)							0.003
	<32	1050	Ref	0.46 (0.20, 1.05)	0.47 (0.24, 0.93)	0.82 (0.43, 1.54)	
	≥32	1314	Ref	1.56 (0.96, 2.52)	3.48 (2.24, 5.41)	1.07 (0.14, 8.40)	
Sodium (mmol/L)							0.008
	<138	1004	Ref	0.84 (0.47, 1.48)	1.54 (0.95, 2.51)	2.23 (1.40, 3.56)	
	≥138	1360	Ref	1.99 (1.09, 3.64)	3.70 (2.16, 6.32)	6.54 (3.87, 11.06)	
Potassium (mmol/L)							0.062
	<4.2	1168	Ref	1.25 (0.70, 2.24)	2.21 (1.34, 3.63)	2.99 (1.85, 4.84)	
	≥4.2	1196	Ref	1.41 (0.79, 2.52)	2.73 (1.64, 4.55)	5.27 (3.20, 8.68)	
SOFA							0.341
	<4	949	Ref	1.10 (0.47, 2.59)	2.67 (1.32, 5.39)	4.17 (2.05, 8.47)	
	≥4	1415	Ref	1.29 (0.80, 2.07)	2.13 (1.40, 3.23)	3.13 (2.10, 4.69)	
SAPS II							0.113
	<38	1160	Ref	2.02 (0.89, 4.58)	3.54 (1.70, 7.34)	5.72 (2.76, 11.83)	
	≥38	1204	Ref	1.00 (0.61, 1.62)	1.85 (1.22, 2.83)	2.61 (1.74, 3.91)	

Binary logistic regression analysis was used and results were presented as OR (odds ratio) and 95% CI (confidence interval). Abbreviation: NPAR, neutrophil percentage to albumin ratio; ACEI, angiotensin-converting enzyme inhibitor; ARB, angiotensin receptor blocker; SOFA, sequential organ failure assessment score; SAPS II, simplified acute physiology score.

**Fig. 5.**
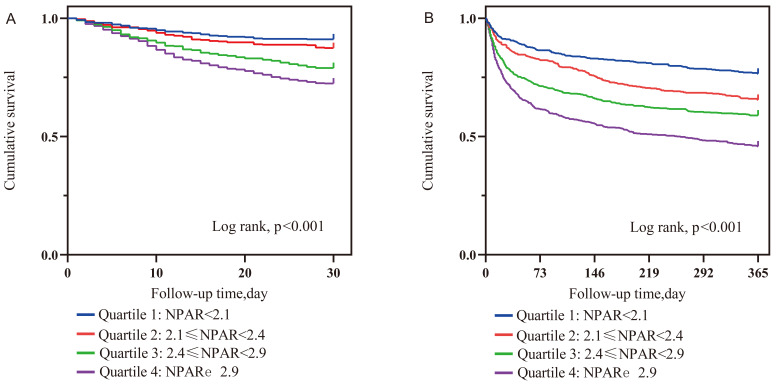
**Kaplan–Meier curves showing the association of NPAR with 30-day (A) and 365-day (B) all-cause mortality**. Abbreviation: NPAR, neutrophil percentage to albumin ratio.

As shown in Table 2, the 30-day (*p *< 0.001) and 365-day (*p *< 0.001) mortality rates, AKI rate (*p *< 0.001), and CRRT rate (*p *< 0.001) increased significantly as the NPAR quartiles increased. The length of CCU (*p *< 0.001) and hospital stay (*p *< 0.001) were prolonged in the higher NPAR quartiles. Kaplan–Meier curves showed that as NPAR quartiles increased, the 30-day (log-rank, *p *< 0.001) and 365-day (log-rank, *p *< 0.001) cumulative survival rates decreased significantly (Fig. 5). In multivariate logistic regression analysis, after adjusting for confounding variables, NPAR was proven to be independently associated with AKI (quartile 4 versus quartile 1: OR, 95% CI: 1.57, 1.19–2.07, *p* = 0.002, *p* for trend = 0.001). However, no significant statistical difference was observed between NPAR quartiles and CRRT (quartile 4 versus quartile 1: OR, 95% CI: 1.46, 0.89–2.41, *p* = 0.133, *p* for trend = 0.044) (Table 6).

**Table 6.** **The association of NPAR with acute kidney injury and renal replacement therapy**.

		Model 1			Model 2			Model 3		
		OR (95% CI)	*p*	p for trend	OR (95% CI)	*p*	*p* for trend	OR (95% CI)	*p*	*p* for trend
Acute kidney injury				<0.001			0.001			0.001
	Quartile 1: NPAR <2.1	Ref			Ref			Ref		
	Quartile 2: 2.1 ≤ NPAR < 2.4	1.25 (0.98, 1.59)	0.073		1.21 (0.95, 1.54)	0.127		1.04 (0.79, 1.37)	0.768	
	Quartile 3: 2.4 ≤ NPAR < 2.9	1.53 (1.22, 1.92)	<0.001		1.50 (1.19, 1.88)	0.001		1.31 (1.01, 1.70)	0.040	
	Quartile 4: NPAR ≥ 2.9	1.87 (1.48, 2.36)	<0.001		1.85 (1.46, 2.34)	<0.001		1.57 (1.19, 2.07)	0.002	
	Continuous	1.42 (1.26, 1.61)	<0.001		1.43 (1.26, 1.61)	<0.001		1.31 (1.14, 1.51)	<0.001	
Renal replacement therapy				0.003		0.002				0.044
	Quartile 1: NPAR <2.1	Ref			Ref			Ref		
	Quartile 2: 2.1 ≤ NPAR < 2.4	1.01 (0.68, 1.50)	0.961		1.02 (0.69, 1.52)	0.914		0.72 (0.41, 1.27)	0.257	
	Quartile 3: 2.4 ≤ NPAR < 2.9	1.44 (1.02, 2.04)	0.040		1.49 (1.05, 2.12)	0.026		1.13 (0.70, 1.85)	0.510	
	Quartile 4: NPAR ≥ 2.9	1.56 (1.10, 2.22)	0.012		1.59 (1.12, 2.27)	0.010		1.46 (0.89, 2.41)	0.133	
	Continuous	1.22 (1.04, 1.43)	0.015		1.23 (1.05, 1.45)	0.010		1.12 (0.89, 1.41)	0.337	

Models were derived from binary logistic regression analysis. Model 1: unadjusted. Model 2: adjusted for age, gender, race. Model 3: adjusted for age, gender, race, systolic blood pressure, diastolic blood pressure, mean blood pressure, respiratory rate, temperature, congestive heart failure, valve disease, cardiogenic shock, hypertension, chronic kidney disease, chronic liver disease, sepsis, beta-blockers, statin, vasopressin, ACEI/ARB, white blood cell, blood nitrogen urea, sodium, creatinine, SAPS II, SOFA. Abbreviation: NPAR, neutrophil percentage to albumin ratio; OR, odds ratio; CI, confidence interval.

## 4. Discussion

The major conclusions drawn can be summarized as follows: (1) As NPAR quartiles increased, in-hospital all-cause mortality increased significantly; even after adjusting for confounding variables, the association between NPAR and in-hospital all-cause mortality remained strong. (2) The results of the ROC curves showed that NPAR had a moderate ability to predict in-hospital mortality in CCU patients. Notably, we found that NPAR was better than PLR and neutrophil count in predicting in-hospital mortality but lower than SOFA and SAPS II. (3) As NPAR quartiles increased, the 30-day and 365-day cumulative survival rates decreased significantly. (4) The Lowess curves presented the non-linear relationship between NPAR and in-hospital mortality. (5) Higher NPAR quartiles were associated with increased AKI and CRRT. After adjusting for possible confounding variables, NPAR was found to be independently associated with AKI. (6) The length of CCU and hospital stay were prolonged as NPAR increased.

Inflammation has been proven to be closely associated with the occurrence, development, and prognosis of coronary atherosclerosis and many other heart diseases. Neutrophils are the most abundant white blood cells in circulation. As effector cells of the natural immune system, neutrophils participate in various immune and inflammatory processes and play an important role in coordinating overall immune and inflammatory responses [15]. Albumin, a classical nutritional marker, is an important transport protein that affects the transport of anti- and pro-inflammatory factors and has antioxidant and anti-inflammatory properties [16]. Several clinical studies have shown that low albumin level is an independent predictor of prognosis in patients with acute coronary syndromes [17, 18]. A low serum albumin concentration is also strongly associated with the development of ischemic heart disease and acute myocardial infarction [19, 20, 21].

As a combination of two classical clinical evaluation parameters, NPAR is an independent predictor of clinical outcomes in many diseases such as septic toxemia, AKI, septic shock, and STEMI [9, 10, 11]. A previous study showed that NPAR, at emergency admission, is an important prognostic indicator of 28-day mortality in patients with severe sepsis [22]. Recent studies in the field of cardiology have shown that NPAR at admission is independently associated with in-hospital mortality in patients with STEMI [23]. For patients with cardiac shock, NPAR is closely associated with in-hospital mortality, 30-day mortality, and 365-day mortality [24]. A study of 3106 patients with extremely severe coronary atherosclerotic heart disease indicated that the risk of all-cause death significantly increased as NPAR increased. After adjusting for confounding variables, NPAR was independently associated with adverse outcomes [25]. Although neutrophil percentage and albumin level have been shown to affect the prognosis of patients with coronary atherosclerosis, NPAR can magnify this change. Clinicians can evaluate the condition more accurately according to NPAR.

Previous studies have confirmed that the inflammation markers, NLR and PLR, have been proven to have a nonlinear relationship with adverse outcomes [26, 27, 28]. The Lowess curve was drawn in our study and the curve revealed J-shaped curves for the relationship between the NPAR and in-hospital mortality, which was consistent with the results of NLR and PLR in previous studies. An inflection point was observed at approximately NPAR = 1.65. From the Lowess curve, we found that NPAR >1.65 was associated with a higher risk of the primary adverse outcome. Notably, when NPAR was <1.65, the mortality rate decreased with an increase in NPAR, suggesting that we should be flexible when using NPAR to judge the disease condition of patients in CCU. When the NPAR value is very small, we should consider whether patients have other comorbidities contributing to increased mortality risk. For example, patients with agranulocytosis have an extremely low NPAR, which has been shown to affect the prognosis of leukemia patients receiving chemotherapy [29]. In this study, we did not exclude patients with hematologic malignancies from hematologic diseases, resulting in lower NPAR values in these patients.

In this study, we compared the influence of NPAR with other clinically common markers such as PLR, NLR, SOFA, and SAPS II. As clinical indicators, PLR and NLR have already been associated with the prognosis of cardiovascular disease [30, 31]. Interestingly, we found that the NPAR was more sensitive in predicting in-hospital mortality in patients in CCU than PLR and NLR. Through the Delong test, SOFA and SAPS II have been demonstrated to be better predictors of adverse outcomes than NPAR. However, NPAR is more cost-effective, can be obtained only through routine admissions, and has a good predictive ability. Especially in cases where a more complex score cannot be calculated, NPAR can replace SOFA and SAPS II as available clinical prognostic factors for critically ill patients.

## 5. Limitation

This was a single-center retrospective cohort study. Due to the limitations of this retrospective study, selection and recall biases could not be avoided, and the causal relationship could not be determined. The failure to dynamically observe the changes in NPAR during hospitalization was also one of the limitations of this study. Although we have done our best to control the bias using multivariate regression, some factors that may affect the model could not be included due to the restriction of the database, such as the left ventricular ejection fraction. Therefore, a multicenter prospective study is required to confirm these findings.

## 6. Conclusions

The NPAR was an independent risk factor for in-hospital mortality in patients in CCU and had a moderate ability to predict in-hospital mortality. As the NPAR quartiles increased, the 30-day and 365-day cumulative survival rates decreased significantly. Also, NPAR was independently associated with AKI, and the length of CCU and hospital stay were prolonged as NPAR increased.

## Data Availability

The data was from MIMIC-III database (https://physionet.org/content/mimiciii/1.4/). Our certificate number is 36571208.
